# The Dynamics of Control: Investigating the Effect of Control Diversity on Subjective Well-Being in Later Life

**DOI:** 10.1093/geroni/igaa057.2082

**Published:** 2020-12-16

**Authors:** Ajit Mann, Jeanne Nakamura

**Affiliations:** Claremont Graduate University, Claremont, California, United States

## Abstract

Recent advancements in research on control beliefs have enabled the assessment of inter-individual differences in its intra-individual variability via a measure of control diversity. However, past research has focused on control diversity in relation to daily stressors. Among a sample of prosocial exemplars, this experience sampling study investigates control diversity across general daily activities and explores its relationship with subjective well-being. Participants indicated the activity they were primarily involved in, the extent to which they felt in control over the situation, and levels of positive and negative affect each time they were signaled; satisfaction with life was measured using a one-time survey at the end of the study. Results from multilevel structural equation modeling suggested that control diversity did not significantly predict positive or negative affect, or satisfaction with life. The study points toward the lack of association between control diversity and subjective well-being and highlights future directions for research.

